# The impact of perceived value and affection on Chinese residents' continuous use intention of mobile health science information: An empirical study

**DOI:** 10.3389/fpubh.2023.1034231

**Published:** 2023-02-08

**Authors:** Kun Tian, Lijie Hao, Wenxia Xuan, Thanawan Phongsatha, Ruimin Hao, Wenjing Wei

**Affiliations:** ^1^College of Digital Arts, Communication University of Shanxi, Taiyuan, China; ^2^Graduate School of Business and Advanced Technology Management, Assumption University, Bangkok, Thailand; ^3^China Australia Business College in Shanxi, Jinzhong, China; ^4^University of Malaya, Kuala Lumpur, Malaysia; ^5^Shanxi Bethune Hospital, Tongji Shanxi Hospital, Shanxi Academy of Medical Sciences, Third Hospital of Shanxi Medical University, Taiyuan, China; ^6^Tongji Hospital, Tongji Medical College, Huazhong University of Science and Technology, Wuhan, China; ^7^Guangzhou Academy of Fine Arts, Guangzhou, China

**Keywords:** health science information, cognition-affect-conation model, Chinese residents, perceived value, affective, continuous use intention

## Abstract

**Introduction:**

Disseminating health science information *via* the internet has become an essential means for improving Chinese residents' health literacy, which has received constant attention from the Chinese government. Therefore, it is important to explore Chinese residents' perceived value and emotional response to mobile health science information for determining Chinese residents' satisfaction and use intention.

**Methods:**

This study applied the cognition–affect–conation model to evaluate the perceived value, arousal, pleasure, trust, satisfaction, and continuous use intention. A mobile device was used to obtain health science information from 236 Chinese residents *via* an online survey and the data were analyzed using partial least squares (PLS)-structural equation modeling.

**Results:**

The results showed that Chinese residents' perceived value of health science information obtained using the mobile device directly affect the degree of arousal (β = 0.412, *P* < 0.001), pleasure (β = 0.215, *P* < 0.01), and trust (β = 0.339, *P* < 0.001). The degree of arousal (β = 0.121, *P* < 0.01), pleasure (β = 0.188, *P* < 0.01), and trust (β = 0.619, *P* < 0.001) directly affected Chinese residents' satisfaction, which further affected their continuous use intention (β = 0.513, *P* < 0.001). Similarly, trust directly affected Chinese residents' continuous use intention (β = 0.323, *P* < 0.001). The degree of arousal directly affected their degree of pleasure (β = 0.304, *P* < 0.001), and pleasure also imposed a direct effect on trust (β = 0.293, *P* < 0.001).

**Discussion:**

The result of this study provided an academic and practical reference to improve mobile health science popularization information. Affective changes have imposed an important effect on Chinese residents' continuous use intention. High-quality, diversified and frequent use of health science information can significantly increase residents' continuous use intention, improving their health literacy as a consequence.

## 1. Introduction

People's health is considered the basic condition of a country's social and economic development and has become an important symbol of a country's prosperity. In 2020, Chinese residents' health literacy rate was 23.15% (1). Due to this relatively low health literacy rate, there was an imbalance in regional development, especially in the prevention and control of chronic diseases and in acquiring knowledge reserved for basic medical treatment (2). The Chinese government has adopted practical measures, such as the promulgation of “China Health 2030” (3), “China Health Action” (4), and other related medical policies and plans, to continuously pay attention to the health of Chinese residents. The rapid development and improvement of Chinese residents' health literacy have provided a good policy environment in China. Although tremendous efforts have been made and a certain level of success has been achieved, Chinese residents' health literacy and status are still not ideal (5). Therefore, it is crucial to improve their health levels by actively improving their health literacy and constructing a holistic, multidimensional, and individualized medical science popularization mode.

Due to the wide application of 5G technology, health science information obtained using mobile devices has become an integral part of people's lives. Mobile health science information changes people's lifestyles and affects Chinese residents' health literacy and behaviors (6, 7). The Chinese government officially released the “Outline of the National Medical and Health System Planning (2015–2020),” which proposed new technologies, such as mobile internet, the internet of things, and cloud computing, to promote Chinese residents' health information services and intelligent medical services (8). Numerous scholars studied the relationships between mobile health science information and health literacy, initiating intensive discussions and drawing attention. Regarding research on mobile health science information, extensive studies focused on the application of information management systems and their implications for physician–patient relationships (9–11). Other studies focused on healthcare information, supplementing and perfecting the existing healthcare system and promoting its future development (12–14). Some scholars indicated that the use of mobile devices for health literacy could promote the dissemination of health science information efficiently and accurately. In addition, some studies showed that mobile health science information provided users with more convenient ways to obtain the necessary information, which greatly improved users' health literacy (15–17). Meanwhile, other scholars found that disseminating health science information in the community could improve Chinese residents' satisfaction (18). Moreover, both online and offline platforms provide users with multiple methods to obtain health science information. Consequently, multiple platforms (WeChat, DouYin, and XiaoHongShu) for health science information have emerged and become the primary ways for Chinese residents to obtain health science information and to improve their health literacy. Although mobile health science information plays a vital role in disseminating health science information, there are still some unresolved questions regarding its use among users and Chinese residents: What is the Chinese residents' perceived value of mobile health science information? How does this perceived value affect their satisfaction and continuous use intention? Mobile health science has become the key to the sustainable development of health science information.

Several studies demonstrated the effectiveness of the cognition–affect–conation model for predicting user behavior. It is widely used in other fields like information and business (19–21). Scholars used this model to predict user stickiness and loyalty. They propose that the ultimate behavior of users is influenced by their intentions, which are closely related to cognition and affection (22, 23). Therefore, the assessment of users' cognition and affection can effectively predict their intentions. Based on this, we used the cognition–affect–conation model to review a few previous literature studies to discover the potential variables that affected Chinese residents' sustainable use of mobile health science information. In addition to enriching the conceptual model of existing research, this study also attempted to build a new conceptual model by investigating the relationship between cognition, affection, and continuous use intention. This study also developed and provided references for the popularization of health science information in the internet era, injecting new driving forces to improve Chinese residents' health awareness and literacy.

## 2. Literature review and hypotheses development

### 2.1. Theoretical background

Human behavior is ordered and purposeful. People carefully consider situations before making decisions (24). In the cognition–affect–conation model (25), the three stages of consumer purchase behavior are proposed: cognitive, affective, and conative, which refer to thinking, feeling, and behaving, respectively. First, consumers begin to realize their needs and collect the necessary information through multimedia platforms. Once consumers know what they need, they progress into the affective stage. In the meantime, the collected information can impose positive or negative emotional feelings, which can influence their consumption. Finally, in the conative stage, consumers behave according to their previous emotions. This theory has been applied and developed in different fields in the internet era. For example, Han and Choi (23) proposed that, in brand consumption, consumers' self-expressed needs were essential to meet their affective experience. The willingness to express their needs inspires consumers' affection, making it efficient to transmit information and promote consumption. Tran et al. (26) researched tourism consumption to explore the relationship between tourists' perceived value and satisfaction as well as the impact on tourists' loyalty to the local tourism brand and their continuous consumption intention. Gao and Lin (27) discovered that consumers' perceived value could be obtained from their cognitive and sensory levels to assess its influence on consumer satisfaction and loyalty. Liang et al. (28) proposed that, in tourism consumption, consumers' perceived service quality and emotional value experience as substantial impacts on their satisfaction and continuous consumption intentions. In the era of information consumption, the use of information has become one of the main consumption methods of consumers. There is a significant relationship between consumers' continuous consumption intention of information and their perceived values (29). An increase in continuous use intention indicates that user stickiness has been enforced and customer loyalty has improved (30). This increase represents the potential value and demand of information by consumers.

Because media use is regarded as social behavior, its value is divided into utilitarian and enjoyment values (31, 32). The practical value is the material needs of consumers for information content, and the enjoyment value is the consumer's spiritual demand for information content. In the consumption process, these two values may overlap and affect changes in consumers' cognition and affection. Emotional perception is the most complicated stage of consumers' psychological activities. At this stage, consumers will not only be affected by the psychological activity of external stimuli beyond information content (33). They can also be affected by their own needs for information content (34, 35). Consumers will have positive and negative emotional changes that are influenced by internal and external factors, ultimately influencing their continuous use intention. However, emotional changes are affected by various factors, such as information quality, trust, pleasure, and satisfaction. This multifactor-influenced relationship is structural and has a progressive relationship between the aforementioned factors (36–38). Therefore, exploring the relationships between the items of affection in the consumption process is essential to users' continuous intention.

Health science information has the characteristics of information consumption. Therefore, it is feasible and effective to use the cognition–affect–conation model as a basis for further research. The model effectively explains an interactive relationship between users' cognition, affection, and conation while exploring how users' continuous use intention is created. In addition, it reveals relationships between the different variables, which significantly influence the improvement and development of health science information.

### 2.2. Hypothesis development

This study explores new variables that influence affection by reviewing a few relevant literature studies on the cognition–affect–conation model. This research also discusses the influencing factors—and their relationships—of Chinese residents' perceived value of health science information and their continuous use intention.

#### 2.2.1. The relationship between perceived value and arousal, pleasure, and trust

Perceived value is users' inner judgment of a product, service, or new technology. It connotes usefulness and comprises dimensions of utilitarian and enjoyment values (31, 32, 39). It has been found that perceived value significantly influences users' affection. Users' affective response has two primary dimensions: valence (negative/positive) and arousal (calmness/excitement) (40, 41). Arousal refers to users' affective level after a specific stimulation (42). When users are stimulated by the information, their emotional states would be aroused, resulting in multiple emotional states (43). Therefore, when Chinese residents use a mobile device to obtain health science information according to their needs, the content will evoke their emotions. As a result, the emotional change influences Chinese residents' assessment of health science information. Perceived value refers to their level of enjoyment, which stimulates their pleasure and satisfaction with products, services, and information (44). Consequently, when Chinese residents apply the necessary health science information, their biological and psychological needs can be met, thus inducing users' pleasantness. Trust significantly influences the relationship and loyalty between users and the use of information (45). Studies showed that high-quality information content provided by mobile service providers (authenticity and credibility) can improve trust and build loyalty among users (46, 47). Therefore, when mobile health science information provides high-quality content, Chinese residents will trust the content, which will strengthen loyalty and user viscosity toward health science information. Therefore, this study outlines the following assumptions based on Chinese residents' perceived value of mobile health science information:

**Hypothesis 1 (H1)**. *Chinese residents' perceived value of mobile health science information significantly influences their arousal*.
**Hypothesis 2 (H2)**. *Chinese residents' perceived value of mobile health science information significantly influences their pleasure*.
**Hypothesis 3 (H3)**. *Chinese residents' perceived value of mobile health science information significantly influences their trust*.

#### 2.2.2. The relationship between satisfaction and arousal, pleasure, and trust

Affective performance is the result of arousal in information processing. A few studies showed that affection assimilates users' evaluation, decisions, and behavior (48, 49). Similar to the manifestation of behavior, satisfaction can also be influenced by affection. Orru et al. (50) proposed that the arousal of affection was the main predictive factor of satisfaction, which confirmed that the level of affection arousal would have various impacts on satisfaction.

The degree of pleasure is a person's good feelings and happiness. Past research confirmed that users' pleasure could affect trust and satisfaction (42, 51, 52). Trust is conceptualized as a set of beliefs about a specific service provider's honesty and ability (53–55). In addition to influencing emotional components (preferences, frequency use, etc.), users' trust also affects use intention (56). Satisfaction is related to the user's emotional composition and use intention. Based on this, the following assumptions were made based on Chinese residents' arousal, pleasure, and trust in mobile health science information:

**Hypothesis 4 (H4)**. *Chinese residents' arousal level in the use of mobile health science information significantly influences satisfaction*.
**Hypothesis 5 (H5)**. *Chinese residents' pleasure level in the use of mobile health science information significantly influences satisfaction*.
**Hypothesis 6 (H6)**. *Chinese residents' trust in the use of mobile health science information significantly influences satisfaction*.

#### 2.2.3. The relationship between satisfaction, trust, and continuous use intention

Users' satisfaction refers to people's expectations and feelings about the use of certain products and services. According to previous studies, users' perceived value directly influenced their use intention. Consistent with the customer satisfaction theory, satisfaction directly affects consumers' willingness to buy products (57). Trust is an essential factor affecting the vigorous development of e-commerce businesses (58). Li et al. (59) and Wang et al. (60) also confirmed that, in the use of information services, there was a relationship between users' trust and their use intention. Consequently, once users' satisfaction and trust in mobile health science information reach a certain level, they will have continuous use intention. Conversely, this intention gradually disappears. According to the abovementioned inference, the following assumptions can be made based on Chinese residents' satisfaction and trust in mobile health science information:

**Hypothesis 7 (H7)**. *Chinese residents' satisfaction with the use of health science information significant influences continuous use intention*.
**Hypothesis 8 (H8)**. *Chinese residents' trust in the use of health science information significantly influences continuous use intention*.

#### 2.2.4. The relationship between arousal, pleasure, and trust

Affection is a kind of attachment produced in the process of cognition. In this process, arousal is an inevitable result of affection (61). A study confirmed a similar arousal-to-pleasure pathway relationship (62, 63). Russell (64) found that pleasure and excitement had an independent dimension in affection. In the arousal process, pleasure can exist as an independent variable. Therefore, we propose that residents' arousal obtained from mobile health science information content can directly affect their pleasure. Eroulu et al. (65) indicated that pleasure had an essential impact on the attitude and behavior of online shoppers. Similarly, in psychology (51) and marketing (52), it was evident that pleasure played an essential role in satisfaction. When a website is used, some users have a high sense of pleasure and continuous access behavior, which means that they are loyal to the website. Here, we define loyalty and trust as consistent when the user's pleasure evokes loyalty. Based on this, we made the following assumptions based on Chinese residents' arousal, pleasure, and trust in mobile health science information:

**Hypothesis 9 (H9)**. *The arousal of Chinese residents' use of mobile health science information significantly influences pleasure*.
**Hypothesis 10 (H10)**. *The pleasure of Chinese residents' use of mobile health science information significantly influences trust*.

### 2.3. Construction of the conceptual model

**Figure 1:**
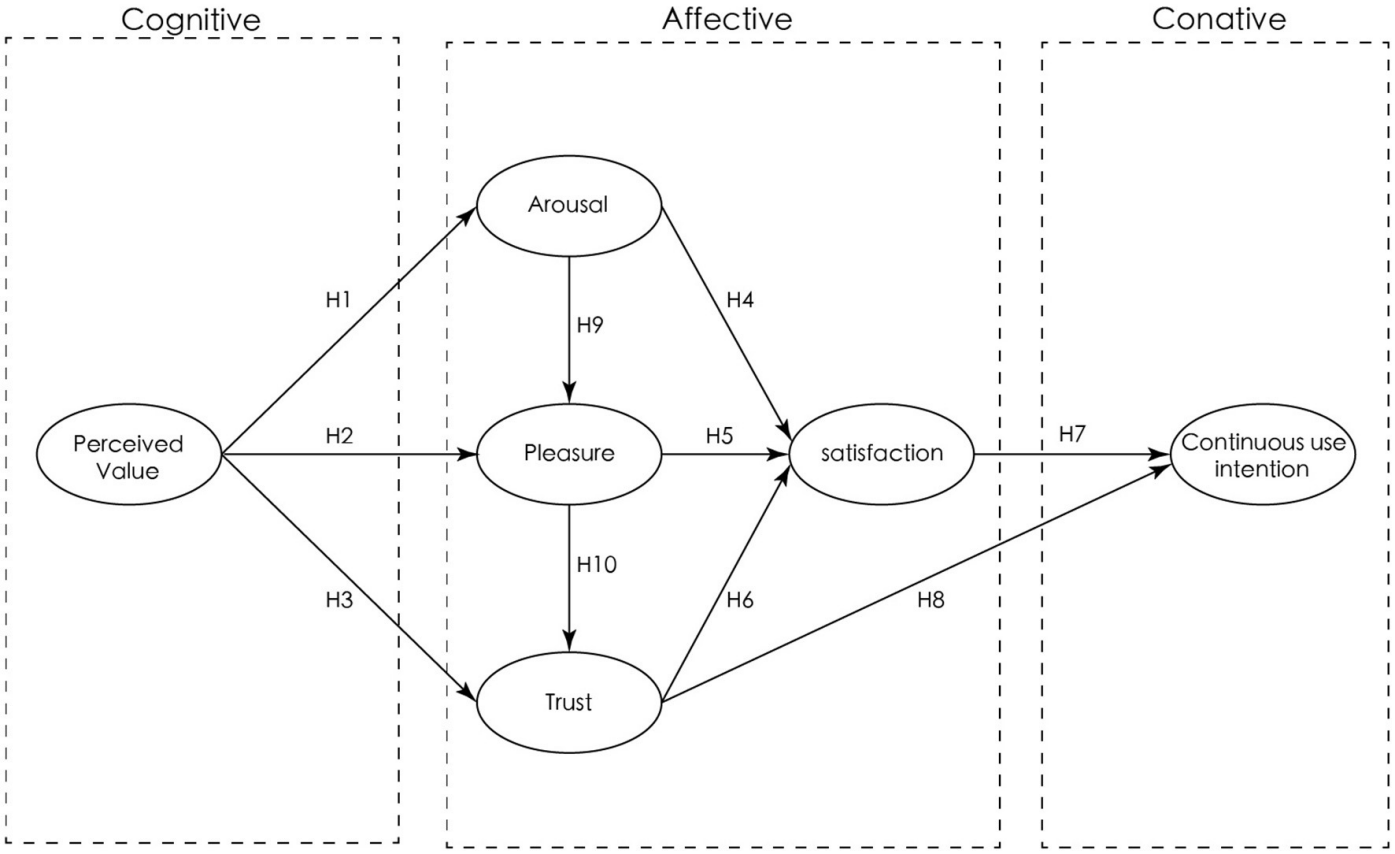
Model construction.

Based on the cognition–affect–conation model, this study intends to explore the influence of factors of Chinese residents' affection on their continuous use intention when they use mobile science information, as shown in Figure 1.

## 3. Methodology

### 3.1. Data collection

The study's questionnaire was designed on the Questionnaire Star System Platform (China). We used a mobile phone to distribute the questionnaire and collect data. Online questionnaires have the following advantages (66): (1) their sampling is not limited to a single geographic location, (2) they are inexpensive, and (3) the responses to them are quicker. To improve participants' willingness and trust, participants were instructed to complete the questionnaire carefully and were randomly allocated rewards ranging from RMB ¥2 to 5 in a red envelope. A total of 308 completed questionnaires were obtained. All participants were from second- and third-tier cities in China to ensure that the data mostly reflect the situation of Chinese urban residents (67). The questionnaires of 45 respondents who did not use mobile phones to read health science information were excluded. Another 27 questionnaires were also excluded because the respondents completed them within <3 min; others in this set had unclear responses. Therefore, 236 valid questionnaires remained, among which 110 were completed by male users, accounting for 46.6% of the total number of participants, and the rest were female users, accounting for 53.4% of the total number of participants.

Regarding age distribution, users aged between 18 and 35 accounted for 24.1% of participants, those aged between 36 and 50 accounted for 38.9%, those aged between 50 and 65 accounted for 29.8%, and those aged over 65 years accounted for 6.7%. Considering participants' educational background, 95.6% of the users had at least a junior high school education, and 93.8% had an income of more than RMB ¥3,000. The demographic characteristics of the data sample indicate that the sample is representative of the Chinese population.

### 3.2. Measurement of variables

The questionnaires used were based on similar scales with high reliability and validity, which were previously used in both domestic and foreign studies. According to Kim et al. (68) and Gupta and Kim (69) research, the perceived value was measured with four question options. The questionnaires in this study were adopted and adjusted from Mehrabian (70) and Watson et al. (71), having three questions respectively to measure arousal and pleasure. According to the studies by Bart et al. (72) and Davis et al. (73), confidence measurement was performed, which had three question options. According to Bhattacherjee (74) and Bhattacherjee et al. (75) study, trust was evaluated and measured with three question options. According to Davis et al. (73) and Cheikh-Ammar and Barki (76), continuous use intention was measured, and three question options were used. The dimensions of the measurement items were set according to the five-point Likert scale, namely, “strongly disagree,” “disagree,” “undecided,” “agree,” and “strongly agree,” with assigned values of 1, 2, 3, 4, and 5, respectively. The items in the questionnaire are presented in Table 1.

**Table 1:** Variable scale and items.

**Variables**	**Factors**	**Items**	**References**
Perceived value	PV1	The content and services provided by mobile health science information are valuable.	(68 , 69 )
	PV2	It is more beneficial to me to read the content of health science information from mobile devices than books, newspapers, Baidu, Toutiao, etc.	
	PV3	Viewing mobile health medical information is more valuable to me than the time I spend checking medical popularization from books, newspapers, Baidu, headlines, etc.	
	PV4	Overall, mobile health science information provides high-quality perceived value	
Arousal	A1	Reading mobile health science information makes you feel (relaxed/ nervous).	(70, 71)
	A2	Reading mobile health science information makes you feel (calm/ excited).	
	A3	Reading mobile health science information makes you feel (tired/ awake).	
Pleasure	P1	Reading mobile health science information makes you feel (angry/ pleasant).	(70, 71)
	P2	Reading mobile health science information makes you feel (unhappy/ happy).	
	P3	Reading health science information makes you (painful/ pleasant).	
Trust	T1	I believe that mobile health science information can solve my concerned question	(72, 73)
	T2	Mobile health science information is helpful to me, and I trust it.	
	T3	The content in mobile health science information is trustworthy.	
Satisfaction	S1	I am satisfied with the overall quality of mobile health science information content	(74, 75)
	S2	I feel satisfied with the content of mobile health science information.	
	S3	I feel satisfied with the service of mobile health science information.	
Continuous use intention	CI1	I will continue to read mobile health science information in the future	(73, 76)
	CI2	I will continue using mobile devices to read health science information even with other platforms.	
	CI3	I will continue reading mobile health science information and increase my reading frequency.	

Because data were collected in China, translation and back translation were performed to ensure accuracy. First, two linguistic professors were consulted to review the significance and readability of each item. The English questionnaire was then translated into Chinese with their assistance. Second, a doctoral student translated the questionnaire from Chinese into English. Third, we compared the translated items to the original English version. To ensure the consistency of the two English versions, we polished the translation and eliminated all inconsistencies.

## 4. Data analysis

This study used partial least squares (PLS) regression to analyze the reliability and validity of data and the estimation and validation of the structural model's path coefficients and explanatory power. Confirming the validity and reliability of variables aids in verifying the relationship between them (77). The PLS method reports causal relationships between the construct variables and deals with the content of model construction and measurement items (78). Concurrently, PLS has relatively loose requirements for the normality and randomness of variables and is suitable for dealing with the relationship between the variables in abnormal data distribution. Furthermore, it can analyze complex predictive models (79). Majchrzak et al. (80) suggested that the maximum number of model paths should be at least 5–10 times the number of samples. The sample size used in this study was 236, and the maximum number of paths was 10, which met the recommended criteria. Therefore, PLS is suitable for analysis. This study used Smart-PLS (Version 3.3.7) developed by Ringle et al. (81).

### 4.1. Reliability and validity test

After analyzing the reliability of the items and the internal consistency, convergent validity, and discriminant validity of each construct, the factor loading of each item in this study met the threshold of 0.6 (82), and the composite of each construct was reliable. The property of composite reliability (CR) value was more than 0.7 (83), indicating that the structure's interior is consistent. In terms of convergent validity, the average variance index (AVE) index of each construct is considered. If the index is >0.5, the construct has good convergent validity (84), as presented in Table 2. According to Table 3, the square root AVE value of all variables is greater than both the value alone and the value of the relationship between this variable and others, indicating that the discriminant validity of the scale is satisfactory.

**Table 2:** Model reliability and validity analysis.

**Variable**	**Item**	**Standardized factor load**	**Cronbach's alpha**	**CR**	**AVE**
Perceived value	PV1	0.703	0.749	0.841	0.571
	PV2	0.791			
	PV3	0.786			
	PV4	0.739			
Arousal	A1	0.735	0.714	0.840	0.638
	A2	0.839			
	A3	0.819			
Pleasure	P1	0.852	0.874	0.922	0.799
	P2	0.930			
	P3	0.897			
Trust	T1	0.845	0.853	0.911	0.773
	T2	0.893			
	T3	0.899			
Satisfaction	S1	0.856	0.829	0.898	0.745
	S2	0.879			
	S3	0.854			
Continuous use intention	CI1	0.878	0.850	0.909	0.769
	CI2	0.874			
	CI3	0.877			

**Table 3:** Square AVE values and correlation coefficients of each variable.

	**A**	**CI**	**P**	**PV**	**S**	**T**
A	0.799					
CI	0.184	0.877				
P	0.392	0.471	0.894			
PV	0.412	0.460	0.340	0.755		
S	0.286	0.744	0.489	0.493	0.863	
T	0.146	0.689	0.408	0.439	0.714	0.879

A, Arousal; CI, Continuous use intention; P, Pleasure; PV, Perceived Value; S, Satisfaction; T, Trust.

The diagonal is the square root of AVE.

In this study, goodness-of-fit (GOF) proposed by Tenenhaus et al. (85) was calculated to know the overall quality of the proposed model, *t*. GOF was calculated as follows:


$$\begin{array}{c} \text{GOF}=\sqrt{\bar{\text{AVE}}\times \bar{R^{2}}}=\sqrt{0.811\times 0.206}\;=\;0.408 \end{array}$$

According to the abovementioned result, GOF was 0.408, which exceeded the cutoff criterion of 0.36 for a large effect size (86). It is shown that the model fit is good.

### 4.2. Hypothesis testing

This study used PLS to test the hypothesized model path coefficients. The hypothetical results are presented in Table 4.

**Table 4:** Summary of hypotheses testing results.

**Hypothesis**	**Path coefficient**	***t*-value**	**Result**
H1: PV → A	0.412^***^	6.453	Supported
H2: PV → P	0.215^**^	3.219	Supported
H3: PV → T	0.339^***^	5.881	Supported
H4: A → S	0.121^**^	2.560	Supported
H5: P → S	0.188^**^	3.187	Supported
H6: T → S	0.619^***^	13.991	Supported
H7: S → CI	0.513^***^	8.616	Supported
H8: T → CI	0.323^***^	5.249	Supported
H9: A → P	0.304^***^	4.201	Supported
H10: P → T	0.293^***^	3.187	Supported

A, Arousal; CI, Continuous use intention; P, Pleasure; PV, Perceived value; S, Satisfaction; T, Trust.

^*^p < 0.05; ^**^p < 0.01; ^***^p < 0.001.

Number of bootstrap samples = 5,000.

**Figure 2:**
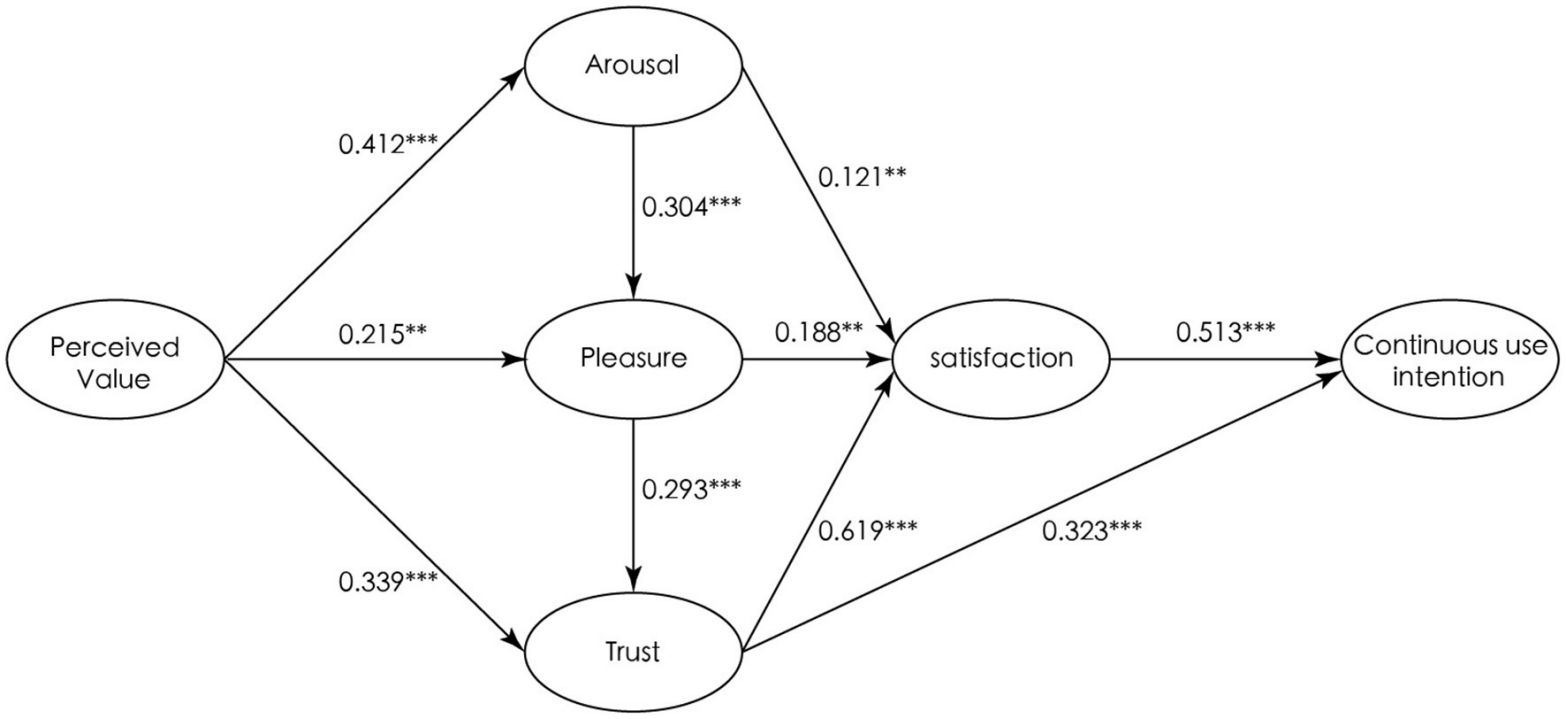
Hypothesis testing results. ^*^p < 0.05; ^**^p < 0.01; ^***^p < 0.001.

In the test of perceived value's effect on arousal, pleasure, and trust, a perceived value significantly positively affected arousal, pleasure, and trust (PV → A: β = 0.412, *t*-value = 6.453; PV → P: β = 0.215, *t*-value = 3.219; PV → T: β = 0.339, *t*-value = 5.881), supporting Hypotheses 1, 2, and 3. The hypothesis test of the effect of arousal, pleasure, and trust on satisfaction revealed that arousal, pleasure, and trust had a significant positive impact on satisfaction (A → S: β = 0.121, *t*-value = 2.560; P → S: β = 0.188, *t*-value = 3.187; T → S: β = 0.619, *t*-value = 13.991), supporting Hypotheses 4, 5, and 6. Satisfaction and trust had a significant positive impact on continuous use intention (S → CI: β = 0.513, *t*-value = 8.616; T → CI: β = 0.323, *t*-value = 5.249), supporting Hypotheses 5 and 6. Hypotheses 9 and 10 in this study were also supported: arousal positively affected pleasure (A → P: β = 0.304, *t*-value = 4.201) and pleasure positively affected trust (P → T: β = 0.293, *t*-value = 3.187). In total, all hypotheses were significantly valid, the model normalized the path (Figure 2).

### 4.3. Testing of mediation effects

This study used the bootstrapping method with bias-corrected confidence estimates to assess the mediation effect (87). We used 95% confidence intervals for specific mediation effects by resampling 5,000 bootstraps. The zero value did not fall within the 95% confidence interval, indicating a significant mediating effect, as presented in Table 5.

**Table 5:** Analysis of the results of the mediation effect.

**Relationship**	**Path coefficient**	**Bias-corrected percentile bootstrap confidence intervals (95%)**
PV → A → S	0.050^*^	(0.018, 0.091)
PV → *P* → S	0.040^*^	(0.014, 0.080)
PV → *T* → S	0.210^***^	(0.140, 0.081)
*A*→*S*→*CI*	0.062^**^	(0.023, 0.106)
*P*→*S*→*CI*	0.097^**^	(0.046, 0.159)
*T*→*S*→*CI*	0.318^***^	(0.253, 0.390)
*PV*→*T*→*CI*	0.110^***^	(0.068, 0.163)
*A*→*P*→*T*	0.089^***^	(0.051, 0.137)

A, Arousal; CI, Continuous use intention; P, Pleasure; PV, Perceived value; S, Satisfaction; T, Trust.

^*^p < 0.05; ^**^p < 0.01; ^***^p < 0.001.

Number of bootstrap samples = 5,000.

## 5. Discussion and conclusions

Based on the cognition–affect–conation theory (25), this study regarded perceived value (cognitive factor) as an independent variable in the theoretical model, affective factor (arousal, pleasure, and trust) as a mediating variable, and satisfaction and continuous use intention as the dependent variables. All these variables are used to build the theoretical model of Chinese residents' continuous use intention of mobile health science information. The empirical results of this study make two important conclusions and contributions. First, Chinese residents' perceived value of mobile health science information can arouse their emotional responses. Changes in their emotional responses can affect their satisfaction and willingness to continue using them. Therefore, this model helps us to understand Chinese residents' expectations and use intentions for mobile health science information and to explore Chinese residents' demand for mobile health science information. Mobile health science information content is an essential factor influencing Chinese residents' continuous use intention. Moreover, the accuracy, timeliness, authority, and expression of the content are the keys to improving the quality of health science information content (15, 16). Therefore, relevant national institutions and mobile internet platforms need to continuously improve the quality of mobile health science information to meet Chinese residents' inherent needs. In addition, the effectiveness of information dissemination can be improved because of the high quality of mobile health science information (88). In this way, health science information can be significantly popularized and promoted among Chinese residents. It was suggested that relevant national institutions and mobile internet platforms should fully understand the core needs of Chinese residents. In addition, health science information should address the most critical issues and dissect its complex, tedious, and challenging content or jargon into comprehensible expressions, allowing Chinese residents to more easily perceive the value of relevant information. Moreover, it is better to enhance the diversity of the content to arouse Chinese residents' interest in health science information. Chinese residents' trust in health science information would be gradually reinforced, satisfaction would improve, and continuous use intention would increase (57, 60).

Second, affection plays a crucial role in decision-making, and the process of affective change is gradual. Changes in affection are affected by many factors such as arousal, pleasure, and trust. In essence, affective change is a change in psychological satisfaction (89). This study found that the affective response of Chinese residents is a process: from arousal to pleasure and finally trust. In this process, Chinese residents' perceived value of mobile health science information affects their affective response of arousal, pleasure, and trust. These factors, in turn, impact their satisfaction and continuous use intention. Therefore, it is evident that the affective response is a complex process, and its subsequent effects are also enormous. In the internet era, the design and promotion of health science information should be guided by the psychological needs of Chinese residents, which should be met progressively.

The previous related studies indicated that content quality, use frequency played important roles in the continuous use intention (56). Provided applied into health science information, these factors should also impose influence on the continuous use intention. Once health science information is regularly and repeatedly implanted into Chinese residents, the stimulation of this content can constantly produce a sense of arousal, pleasure, trust, and satisfaction. By this method, health science information can be fully understood and used by residents.

## 6. Research limitations

In this study, despite strict controls on research design, methods, and data collection, some limitations remain to be addressed in future studies. First, because the data sample in this study is cross-sectional, the analysis results can only explain current Chinese residents' willingness to use mobile health science information. Thus, long-term user intention and willingness need further longitudinal observation. Second, although the use of mobile devices to promote health science information has gradually become a mainstream method, offline communication is also indispensable. Therefore, future research should test the model in both online and offline scenarios. Third, because of time and space constraints, samples in this study were selected only from second- and third-tier cities in China, which inhibited generalization to the entire Chinese population. Thus, more cities and regions, such as developed first-tier cities and underdeveloped remote rural areas, should be incorporated into future research, which will expand the sample size, further improve the accuracy of the research, and allow for generalization. Finally, in future research, it is possible to test whether the characteristics of Chinese residents (income, educational level, health concerns, etc.) influence their continuous use intention of mobile health science information.

## Data availability statement

The raw data supporting the conclusions of this article will be made available by the authors, without undue reservation.

## Author contributions

KT wrote the draft of the manuscript. WX contributed to the samples of the research. LH played a major role in the technical support. RH and WW gave support to the data analysis. TP gave guidance on the methodology. All authors contributed to the article and approved the submitted version.
